# A Comparative Study to Check the Accuracy of Tooth Shade Selection With Standardized Digital Photographs and a Spectrophotometer

**DOI:** 10.7759/cureus.56073

**Published:** 2024-03-13

**Authors:** Malik Hina, MD Sadique Ali, Deepmala Pande, Kaushik Kumar Pandey, Nikhil Kandwal, Doyir Tasar, Monika S Jadhav, Sruthy Xavier, Gokulkrishna S

**Affiliations:** 1 Prosthodontics, Career Post Graduate Institute of Dental Sciences and Hospital, Lucknow, IND; 2 Prosthodontics, Saraswati Dental College, Lucknow, IND; 3 Prosthodontics, Hitkarini Dental College and Hospital, Jabalpur, IND; 4 Prosthodontics, Uttaranchal Dental and Medical Research Institute, Dehradun, IND; 5 Prosthodontics, Sriram Chandra Bhanja Dental College and Hospital, Cuttack, Cuttack, IND; 6 Maxillofacial Prosthodontics and Oral Implantology, Mahatma Gandhi Vidyamandir's Karmaveer Bhausaheb Hiray Dental College and Hospital, Nashik, IND; 7 Prosthodontics, Government Dental College, Kottayam, Kottayam, IND

**Keywords:** tooth shade matching, shade matching, aesthetics, spectrophotometer, digital photography, shade selection

## Abstract

Background: The success of any dental prosthesis depends on aesthetics and function. A proper shade selection is a prime requisite for providing aesthetics to dental patients.

Purpose: This study aims to compare the photographic method of shade selection with that of a digital spectrophotometer.

Materials and methods: The study included 50 participants. The primary inclusion criterion was the presence of the maxillary left central incisor without a history of restorative or endodontic procedures. The shade of the left maxillary central incisor was determined using the VITA Easyshade V spectrophotometer (VITA Zahnfabrik, BadSäckingen, Germany) and the digital photography method for all the selected participants. The CIELAB colour space utilizes three values (L*, a*, and b*) to objectively measure colour. While the digital photography approach used Adobe Photoshop software (Adobe Systems Incorporated, San Jose, CA) to report solely the L*, a*, and b* values, the spectrophotometer reported the L*, a*, and b* values along with the actual shade. After obtaining the values of L*, a*, and b*, ΔE, which is their difference, was calculated using a standard formula. Statistical analysis was carried out by using Student's t-test and proportion z-test.

Results: When the comparison of the L*a*b* values was done, Student’s t-test showed similar (P>0.05) L(t=0.16, P=0.872), a (t=0.52, P=0.607), and b (t=0.23, P=0.820) values between the two groups; that is, they did not differ significantly or showed perfect matching. There was agreement (ΔE≤2) in 42 (84.0%) cases and disagreement (ΔE>2) in eight (16.0%) cases. The proportion z-test showed an agreement of 84.0%, which was statistically highly significant (z=20.44, P<0.001).

Conclusion: The true shade of the teeth can be depicted using standardized digital images.

## Introduction

Colour is of critical importance to the outcome of dental restorations and their acceptance by patients. A rise in patient consciousness has resulted in an expansion of the market for aesthetic restorations. The second most common reason for the remake of restoration is an unmatched shade, with the first being the tooth preparation and impression. An unnatural appearance of the restoration is a key factor that leads to its replacement [1].

The instrumental method was introduced in the late 1990s to overcome the imperfections and inconsistencies of traditional shade matching. The most common devices are colourimeters and spectrophotometers, which calculate the tooth's colour by measuring the amount and spectral composition of reflected light on the tooth's surface. Objective readings and quicker results are some of the benefits of these methods [2]. In dentistry, spectrophotometers are highly precise, useful, and versatile instruments for colour matching [3]. Spectrophotometers use the CIELAB system, where L* represents lightness, while the a* and b* values indicate position on the red/green and yellow/blue axes, respectively. It was discovered that spectrophotometers provide a 33% increase in accuracy and a more objective match in 93.3% of cases compared to observations made by the human eye or traditional techniques [4]. However, spectrophotometers are not widely used in clinical practice due to their high cost. One disadvantage of spectrophotometers is that they lose a portion of the light that enters the tooth, known as the "edge-loss error" [5].

Digital photography is a powerful medium of case documentation and communication. Photographs make it possible to evaluate several points on a tooth, which can aid in determining the true shade using appropriate imaging software. The advantages include objectivity, lower cost, convenience, the ability to evaluate several points on a tooth, easier patient record maintenance, and efficient communication with the dental laboratory. The images are simple to store and are easily transferable via electronic mail for consultation. It is however not without limitations. The image quality depends on the camera and the lens used, and the colour cast is a concern that needs to be eliminated [5]. Hence, this study was planned to compare the digital photographic method with the spectrophotometer for the evaluation of teeth shade.

## Materials and methods

This study was conducted at the Department of Prosthodontics and Crown and Bridge at Career Postgraduate Institute of Dental Sciences and Hospital, Lucknow, India, between the years 2019 and 2020. The study proceeded after acquiring the necessary approval from the ethical committee of the institution (CPGIDSH/22/57). A total of 50 subjects were included in this study. The age range of all subjects was between 18 and 45 years. Armamentarium used in the study were the following: Canon 200D SLR camera (Canon, Tokyo, Japan), Tokina 100mm 2.8F macro lens (Kenko Co., Ltd, Japan) with Yongnuo YN24EX macro flash 18% grey card, VITA Easyshade V digital spectrophotometer (VITA Zahnfabrik, BadSäckingen, Germany), and Hp Pavilion x360 (Core i7, 12 GB RAM) with Adobe Photoshop CC software (Adobe Systems Incorporated, San Jose, CA). Subjects were selected according to the following inclusion and exclusion criteria.

Inclusion criteria: Subjects having an intact maxillary left central incisor with no history of restorative or endodontic procedures.

Exclusion criteria: Patients are to be excluded if the concerned tooth had the following: a history of trauma, discolourations, white spots, composite fillings, veneers or crowns, and endodontic treatment.

Methods

Shade Selection by the VITA Easyshade V Spectrophotometer

**Figure 1:**
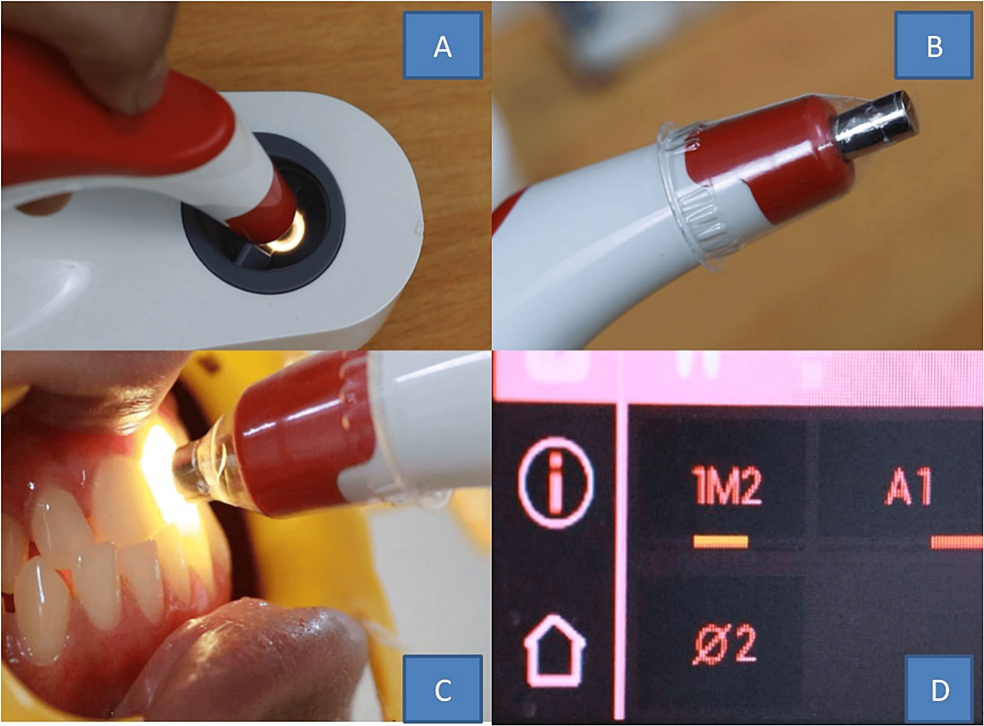
Shade taking with a spectrophotometer. A) Calibration of the device, B) infection control shield, C) device probe set at right angles, and D) shade display in classical & 3D Master systems

The shade was taken using VITA Easyshade V with ‘tooth single program’ selected. The gadget was calibrated, and shade recordings were made in accordance with the manufacturer's instructions. After calibrating the device, the infection control shield was snapped onto the device probe. The central region of the labial surfaces of maxillary left central incisors was measured with the device probe set at right angles. The measurement result was shown in the VITA 3D Master and A1-D4 VITA classical tooth shade systems. The reading was taken several times until three constant readings were achieved, and this reading was noted. The L* a* b* values are also noted. The plastic tip is changed after each use, and device calibration is done before taking each reading (Figure 1).

Shade Determination Using Intra-oral Photographs (DSLR)

**Figure 2:**
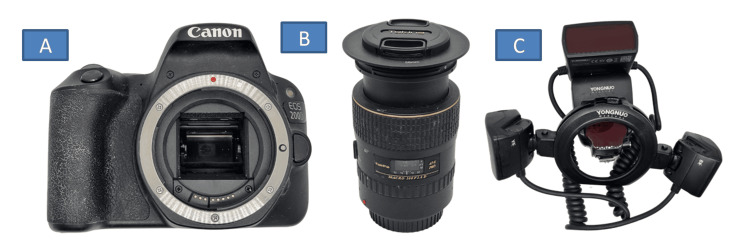
DSLR camera with attachments used for photographic shade taking. A) Canon 200D SLR Camera, B) Tokina 100mm 2.8F macro lens, and C) Yongnuo YN24EX macro flash

**Figure 3:**
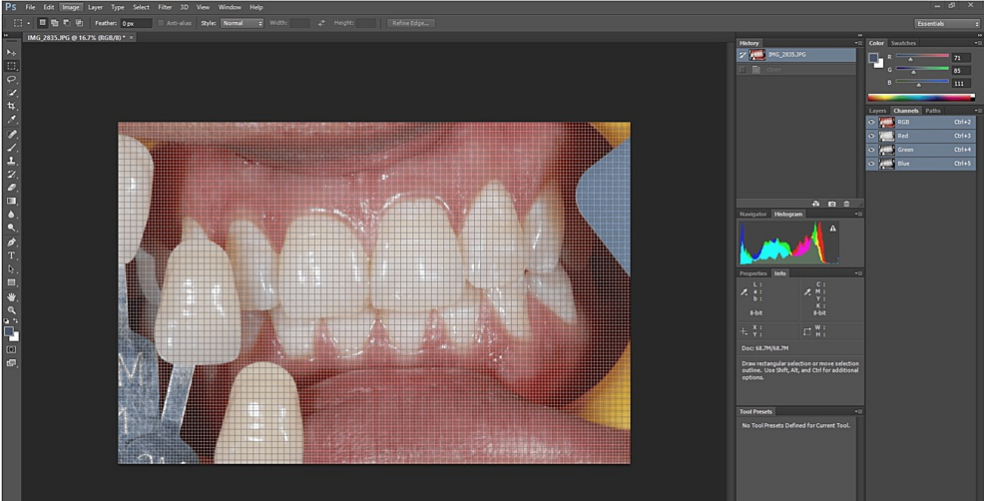
Determination of L* a* b* values using Abode Photoshop.

Digital photographs were taken with Tokina 100mm 2.8F macro lens with Yongnuo macro flash mounted on a Canon 200D SLR camera, at a constant distance by maintaining a fixed magnification ratio of 1:3. To standardize the colours, a grey card with an 18% shade was placed inside the tooth. Adobe Photoshop CC software was utilized for this purpose, as depicted in Figures 2-3. The saved image was in JPEG format. The JPEG image was opened in Adobe Photoshop by pressing "CTRL+O" and processed following the protocol defined by Bengel [6]. After opening the image, "CTRL + L" was pressed to remove any colour cast. Alternatively, Image > Adjust > Levels could also be used. This brought up a histogram and three eye-dropper tools, including three dropper tools. Placing the middle dropper tool over the grey card in the image resulted in the RGB (red, blue, and green) values. As spectrophotometers provide L* a* b* values, it was necessary to convert RGB to L* a* b*. This was done by clicking on the "image" option in the main toolbar, followed by "mode," and then selecting L* a* b* colour. The L* a* b* values of the grey card were obtained in this manner.

The grey card used in the photographic method had known L* a* b* values of 54, 0, and 0. To standardize the image, the brightness level was adjusted to an L value of 54 by clicking Image, Adjust, and then Brightness. This sets the brightness of the entire image to a fixed value. To isolate the maxillary left central incisor, the magnetic lasso tool was used. The "Magic Wand" tool was then utilized to remove the reflection on the tooth surface. Once isolated, the L* a* b* values of the tooth were determined and depicted in the Photoshop histogram. The software-derived L* a* b* values were then converted to the L* a* b* values as given by the CIEL* a* b* system using the following formula: • L* = L(PM) × 100/255 • a* = a(PM) − 128) × 240/255 • b* = b(PM − 128) × 240/255

L(PM), a(PM), and b(PM) refer to L* a* b* values derived from Adobe Photoshop.

From the above formula, L* a* b* values as per the Commission Internationale de l'Eclairage (CIE) were thus recorded for each subject. The difference in the colour shade (ΔE) was determined by comparing the L* a* b* values obtained from the spectrophotometer and digital photography. The calculation formula used for this purpose was ΔE = [(L1 − L2)² + (a1 − a2)² + (b1 − b2)²]1/2.

The numbers obtained from the spectrophotometric measurement are L1, a1, and b1. The values that were acquired using the digital photography technique are L2, a2, and b2. For ΔE<2 and more than 2, a score of "agreement" or "not in agreement" is assigned, respectively. This is done because, as proposed by Della Bona et al. [7], the hue difference (ΔE) of < 2 between two objects is not visible to the human eye. Student’s t-test was used to compare the mean L* a* b* values between the two methods, and the proportion z-test was used to compare the ΔE values.

## Results

The readings were tabulated and subjected to statistical analysis using Student's t-test and proportion z-test. The L* a* b* values of the spectrophotometer and digital photographs are shown in Tables 1-2, respectively. The L1, a1, and b1 values (spectrophotometric values) of dentate patients ranged from 68.4 to 87.8, -3.7 to 3.6, and 8.9 to 33.1, respectively, with a mean ± SE of 79.26 ± 0.68, -0.58 ± 0.25, and 16.91 ± 0.75, and a median of 79.1, -1.0, and 16.0, respectively. The L2, a2, and b2 values (photographic values) of dentate patients ranged from 70.2 to 88.1, -4.1 to 3.9, and 9.3 to 32.2, respectively, with a mean ± SE of 79.41 ± 0.65, -0.76 ± 0.26, and 17.15 ± 0.71, and a median of 79.0, -1.3, and 16.2, respectively. Comparing the mean L*, a*, and b* values between the two methods (Table 3 ), Student’s t-test showed similar values (P > 0.05), that is, L* (t=0.16, P=0.872), a* (t=0.52, P=0.607), and b* (t=0.23, P=0.820), between the two groups (i.e., did not differ significantly or showed perfect matching). Of the total 50 cases, both methods showed agreement (ΔE≤2) in 42 (84.0%) cases, whereas disagreement (ΔE>2) in eight (16.0%) cases. The proportion z-test showed an agreement of 84.0% that was statistically highly significant (z=20.44, P<0.001) (Table 4).

**Table 1:** The L* a* b* values of dentate patients in spectrophotometer are summarized in range (min to max), mean ± SE, and median.

L* a* b* values	Range (min to max)	Mean ± SE (n=50)	Median
L1	68.4 to 87.8	79.26±0.68	79.1
a1	-3.7 to 3.6	-0.58±0.25	-1.0
b1	8.9 to 33.1	16.91±0.75	16.0

**Table 2:** The L* a* b* values of dentate patients in the digital photographic method are summarized in range (min to max), mean ± SE, and median.

L* a* b* values	Range (min to max)	Mean ± SE (n=50)	Median
L1	70.2 to 88.1	79.41±0.65	79.0
a1	- 4.1 to 3.9	-0.76±0.26	-1.3
b1	9.3 to 32.2	17.15±0.71	16.2

**Table 3:** L* a* b* values of the two groups are summarized in mean ± SE and compared by independent Student’s t-test (t value).

L* a* b* values	Spectrophotometer (n=50)	Digital photographic method (n=50)	Mean difference	T value	P value
L1	79.2±60.68	79.41±0.65	0.15±0.94	0.16	0.872
a1	-0.58±0.25	-0.76±0.26	0.19±0.36	0.52	0.607
b1	16.91±0.75	17.15±0.71	0.24±1.03	0.23	0.820

**Table 4:** Distribution of the overall agreement of ΔE values (difference in the teeth shade) between spectrophotometer and digital photographic methods in dentate patients (n=50). The proportion z-test showed an agreement of 84.0% that was statistically highly significant (z=20.44, P<0.001).

Agreement –ΔE values	Spectrophotometer vs digital photography (n=50) (%)
>2 (No)	8 (16.0)
≤2 (Yes)	42 (84.0)

## Discussion

Over the past few decades, there has been a growing demand for aesthetic restorations in the field of dentistry. In the past, dental restorations made of gold, such as gold fillings and crowns, were seen as a status symbol and were popular among many patients. But now, the goal is to achieve a natural look that mimics the patient's teeth. This means that the restored teeth must be accurate in terms of form, translucency, texture, and shade to create an aesthetically pleasing result. Even a slight mismatch between the restoration and the patient's natural teeth can lead to costly refabrication or replacement of the restoration, which can negatively affect the dentist's reputation [8].

The methods of shade selection can be broadly classified into conventional and digital techniques. Conventional methods include shade guides, while digital methods include spectrophotometers, colourimeters, and film-based and digital photography [9,10].

Instrumental colour analysis overcomes many of the disadvantages of visual shade determination because the colour determination of the former method is objective, can be quantified, and is more rapidly obtained [2]. Spectrophotometers operate with a standardized internal light source; that is, unlike the visual method, it is unaffected by extrinsic factors. Its accuracy however depends on the instrument used, type of material, opacity, translucency, and texture of the measured area [11]. Horn et al. in 1998 in an in-vitro study concluded that spectrophotometry is a more reliable and predictable way of assessing tooth shade compared to the human eye [12]. Paul et al., in 2002, made similar conclusions about the precision and reproducibility of spectrophotometers. However, owing to the cost and their complexity, spectrophotometers and colourimeters have been used mainly in research and are less common in clinical practice [4].

To overcome the limitations of the above methods, there is a need for an alternative approach that is accurate and cost-effective and serves the purpose of being a tool for communication. Here, standardized digital photographs can potentially serve the purpose. Digital cameras have multiple uses, such as dental documentation, treatment monitoring, communication, and marketing promotion [13,14].

Bengel in 2003 stated that digital photographs are influenced by light and camera technology. They proposed the use of a grey card as a neutral reference object within the image frame that can eliminate the colour cast of the image by the use of software such as Adobe Photoshop [6]. The best way to communicate colour is by reference photography using digital camera-obtained reference shade tabs from existing shade guide systems, according to a 2010 review by Chu et al. [5].

VITA Easyshade V was used in this study, a gold standard in spectrophotometers. A Canon 200D SLR camera was used with a 100mm macro lens, which is considered the ideal lens for macro photography. Because of its flash setup, twin flash was used because it is ideal for esthetic case documentation.

The L* a* b* values obtained from the spectrophotometer and photographs can be found in Tables 1-2, respectively. As shown in Table 3, a comparison of these L* a* b* values was performed, and Student's t-test revealed no significant difference (P>0.05) in L (t=0.16, P=0.872), a (t=0.52, P=0.607), and b (t=0.23, P=0.820) values between the two groups. This indicates that the results were similar and matched perfectly. The colour difference between two objects expressed as ΔE is presented in Table 4. There was agreement (ΔE≤2) in 42 (84.0%) cases and disagreement (ΔE>2) in eight (16.0%) cases. The proportion z-test showed an agreement of 84.0%, which was statistically highly significant (z=20.44, P<0.001). These results are in accordance with a study conducted by Miyajiwala et al., who found ΔE agreement in 31 (62%) cases and disagreement in 19 (38%) cases. On comparing the agreement using z‑test for proportions, the results were statistically significant with a higher proportion of “yes” (agreement) (z=-3.2 and P=0.00138 (P<0.01) [14].

These results are in accordance with a study conducted by Miyajiwala et al., who found ΔE agreement in 31 (62%) cases and disagreement in 19 (38%) cases. When the agreement was compared using the proportions z-test, the results showed a higher proportion of "yes" (agreement) (z=-3.2 and P=0.00138 (P<0.01)), which was statistically significant [14].

Anand et al. [15] investigated if digital pictures captured by an SLR camera could serve as a substitute for the VITA Easyshade spectrophotometer in determining the shade of teeth. The study established that the L* a* b* values acquired through both methods had a significant correlation, except for the a value, which was not significant. Consequently, it was concluded that an SLR camera, with Adobe Photoshop CS5.1 as an adjunct, could be utilized as an alternative to a spectrophotometer for accurately obtaining the L* and b* values [15].

The present study has shown a statistically significant result due to the high level of standardization for capturing photographs. The images were taken under controlled environmental factors with an 18% grey card kept within the image frame. The grey card has its red, blue, and green values equal, so it serves as a neutral shade [16,17]. A 100mm macro lens was used in manual mode, and the ideal settings for the exposure triangle were kept as follows: ISO: 100, shutter speed: 1/200, F: 22, and magnification ratio: 1:3. A Yongnuo twin flash was used to illuminate the pictures. It has two light sources that illuminate the images from its side. Thus, it directs the light to all corners of the mouth without reflecting. This is an advantage for shade selection. After capturing the images, their L* a* b* values were analyzed in Adobe Photoshop CC. The software treated the grey card as a reference for correcting the colours of the entire image [15].

Delta E (ΔE) measures the difference between two colours. The smaller the value of ΔE, the more similar the two colours are. A ΔE value of zero means that the colours match perfectly, and it cannot be detected by the human eye. On the other hand, the higher the value of ΔE, the further away the colour is from the true hue, using the CIELAB colour space. The colour difference that can be detected at a minimum is typically between 1 and 2.5 ΔE, as per a few studies. A ΔE ranging from 3 to 6 is deemed satisfactory for commercial reproduction, but printing and graphic professionals might still note a difference in colour perception [4,18,19].

The potential limitations of this study are as follows: (1) The participants were confined to a limited geographic area. (2) Male and female samples were not equally taken, so this study did not give suitable results for a particular gender.

## Conclusions

Within the limitations of this study, it can be concluded that standardized digital photographs can capture correct teeth shades. Digital photographs can serve as an effective tool for shade matching. It is a versatile device used for record-keeping, communication, dental marketing, and trust-building, and following the correct protocol, it can help access the correct shade of the teeth.
